# Virucidal Properties of Photocatalytic Coating on Glass against a Model Human Coronavirus

**DOI:** 10.1128/spectrum.00269-22

**Published:** 2022-05-04

**Authors:** Ángel L. Álvarez, Kevin P. Dalton, Inés Nicieza, Fabio A. Abade Dos Santos, Pilar de la Peña, Pedro Domínguez, José M. Martin-Alonso, Francisco Parra

**Affiliations:** a Departamento de Bioquímica y Biología Molecular, Instituto Universitario de Biotecnología de Asturias, Universidad de Oviedo, Oviedo, Spain; b Instituto Nacional de Investigação Agrária e Veterinária, Oeiras, Portugal; University of Georgia

**Keywords:** virucidal activity, human coronavirus, photocatalytic coating, titanium dioxide

## Abstract

The antimicrobial properties of photocatalysts have long been studied. However, most of the available literature describes their antibacterial properties, while knowledge of their antiviral activity is rather scarce. Since the outset of the coronavirus disease 2019 (COVID-19) pandemic, an increasing body of research has suggested their antiviral potential and highlighted the need for further research in this area. In this study, we investigated the virucidal properties of a commercial TiO_2_-coated photocatalytic glass against a model human coronavirus. Our findings demonstrate that the TiO_2_-coated glass consistently inactivates coronaviruses upon contact under daylight illumination, in a time-dependent manner. A 99% drop in virus titer was achieved after 3.9 h. The electron micrographs of virus-covered TiO_2_-glass showed a reduced number of virions compared to control glass. Morphological alterations of TiO_2_-exposed viruses included deformation, disruption of the viral envelope, and virion ghosts, endorsing the application of this material in the construction of protective elements to mitigate the transmission of viruses. To the best of our knowledge, this is the first report showing direct visual evidence of human coronaviruses being damaged and morphologically altered following exposure to this photocatalyst.

**IMPORTANCE** Surface contamination is an important contributor to SARS-CoV-2 spread. The use of personal protective elements and physical barriers (i.e., masks, gloves, and indoor glass separators) increases safety and has proven invaluable in preventing contagion. Redesigning these barriers so that the virus cannot remain infectious on them could make a difference in COVID-19 epidemiology. The introduction of additives with virucidal activity could potentiate the protective effects of these barriers to serve not only as physical containment but also as virus killers, reducing surface contamination after hand touch or aerosol deposition. We performed in-depth analysis of the kinetics of photocatalysis-triggered coronavirus inactivation on building glass coated with TiO_2_. This is the first report showing direct visual evidence (electron microscopy) of coronaviruses being morphologically damaged following exposure to this photocatalyst, demonstrating the high potential of this material to be incorporated into daily-life high-touch surfaces, giving them an added value in decelerating the virus spread.

## INTRODUCTION

Since the outset of the coronavirus disease 2019 (COVID-19) pandemic, its etiological agent—severe acute respiratory syndrome coronavirus 2 (SARS-CoV-2)—has infected more than 445 million individuals worldwide and led to more than 5.9 million deaths (1). In the wake of the pandemic, virology laboratories and pharmaceutical companies all around the globe have rallied for vaccines, with 342 candidates currently in preclinical or clinical trials and more than 20 vaccines already approved for emergency use in several countries (2).

Like most respiratory pathogens, SARS-CoV-2 can spread directly from an infected person’s mouth or nose in small liquid particles when they cough, sneeze, speak, breathe, or undergo medical procedures. These particles range from large respiratory droplets to small aerosols. It is generally accepted that the large droplets are too heavy to remain airborne and ultimately fall, further contaminating the surfaces below (3). Additionally, infected individuals can self-contaminate their hands and contaminate public railings, door handles, elevator buttons, and other high-touch surfaces. Virus transmission via indirect contact takes place when an uninfected individual touches a contaminated surface and self-inoculates their own mucous membranes, such as the ocular, nasal, and oral mucosae (4).

Thus, in addition to the unprecedented fast vaccine response, the pandemic also brought to the fore the importance of environmental hygiene and cleaning procedures to control virus dissemination. The currently recommended nonpharmaceutical approaches to minimize transmission include social distancing, the wearing of face masks, handwashing, and frequent use of disinfectants. In this sense, the accumulating evidence of nonaerosol virus transmission among COVID-19 cases (5) highlights the need for further research to understand the mechanisms underlying virus survival or inactivation on surfaces, as well as the development of novel materials and coatings with virucidal properties, which could be a very effective method to mitigate the spread of the pandemic (5).

The antimicrobial properties of photocatalytic materials have been intensely studied. Some of the most prominent antimicrobial photocatalytic materials are TiO_2_-based coatings. Upon excitation by light, the photon energy generates an electron-hole pair on the TiO_2_ surface, a highly unstable state with strong oxidation/reduction power that converts water into hydroxyl radicals (^·^OH), superoxide ions (O_2_^·−^) and hydrogen peroxide (H_2_O_2_). These reactive oxygen species (ROS) are hypothesized to exert their microbicidal action through oxidative stress, causing nucleic acid damage, lipid peroxidation, and biological membrane disruption (6; reviewed in reference 7). In this study, the antiviral effects of commercial photocatalytic TiO_2_-coated glass were analyzed. The coating consists of a transparent and permanent layer of nanoscale TiO_2_ on glass with self-cleaning capability thanks to its photocatalytic and hydrophilic properties. We aimed to investigate the ability of this material to physically inactivate virus particles, namely, those of human coronavirus 229E (HCoV-229E), as well as measuring the kinetics of virus survival upon contact with this material.

## RESULTS AND DISCUSSION

Human coronavirus 229E (HCoV-229E) is a pathogen responsible for seasonal respiratory illness outbreaks (family *Coronaviridae*, genus *Alphacoronavirus*, subgenus *Duvinacovirus*). HCoV-229E virus has been widely used in coronavirus antiviral research. When exposed to different surface materials, it shows survival patterns similar to those of SARS-CoV and SARS-CoV-2 (8), and it was recently validated as a good surrogate model for SARS-CoV-2, due to their morphological, physicochemical, and biological similarities (9).

In this study, we sought to evaluate the virucidal activity of a TiO_2_-based photocatalytic coating on glass and investigate whether the illumination of this material before and/or during incubation with the virus influences its virucidal capability. UV-A light was used to photocatalytically preactivate the glass for 4 h prior to virus inoculation, and its virucidal effects were further assessed in the presence of D65 light, a type of radiation that emulates daylight. For this purpose, a commercial luminaire capable of emitting D65 light was assembled inside the biosafety class II cabinet, allowing safe handling of the potentially pathogenic human coronavirus 229E, as shown in Fig. S1A (top).

The virucidal assay consisted of inoculating a fixed amount of virus on the glass in the form of evenly distributed small droplets, as shown in Fig. S1A (lower left), and further covering the virus inoculum with a high-transmittance polyvinyl chloride (PVC) film (Fig. S1A, lower right). Each glass assembly was further incubated at room temperature for a specific interval according to the study time course. The infectious titer reduction (virucidal activity) due to virus contact with the TiO_2_-coated material was determined by 50% endpoint titration (50% tissue culture infective dose [TCID_50_] infectivity assays), where viruses exposed to TiO_2_ were compared to an equivalent virus inoculum exposed to bare (control) float glass for an equivalent interval.

Most infectivity assays rely on the ability of viruses to morphologically alter the infected cell monolayer (cytopathic effect [CPE]). These alterations may include but might not be limited to cell rounding and detachment, loss of monolayer integrity (confluence), appearance of cytosolic and/or nuclear inclusion bodies, refractivity, etc. (Fig. S1B, upper right). However, such alterations are often ambiguous, hard to identify, or difficult to distinguish from other morphological changes unrelated to infection, such as normal cell senescence, rendering this assay highly subjective. In our work, we overcame this limitation of conventional TCID_50_ assays by taking advantage of a recombinant green fluorescent protein (GFP)-expressing human coronavirus 229E strain. Since the host cells do not harbor the GFP coding sequence, any single green-fluorescent cell in the monolayer is considered virus infected, and the corresponding well in the plate is marked as positive during the titer calculation (Fig. S1B, lower right). While the absence of visually detected CPE (Fig. S1B, top left) does not necessarily imply the lack of infection (e.g., CPE is absent at early stages of infection), the lack of green-fluorescent cells unambiguously indicates the absence of GFP expression and, thus, the absence of virus infection (Fig. S1B, lower left).

Three different illumination conditions were separately assayed: the virucidal experiment was performed on test surfaces in the absence of light, in the presence of D65 following preactivation with UV-A light, and in the presence of D65 without previous activation with UV-A light.

### Photocatalysis is required for TiO_2_-mediated virucidal activity.

**FIG 1:**
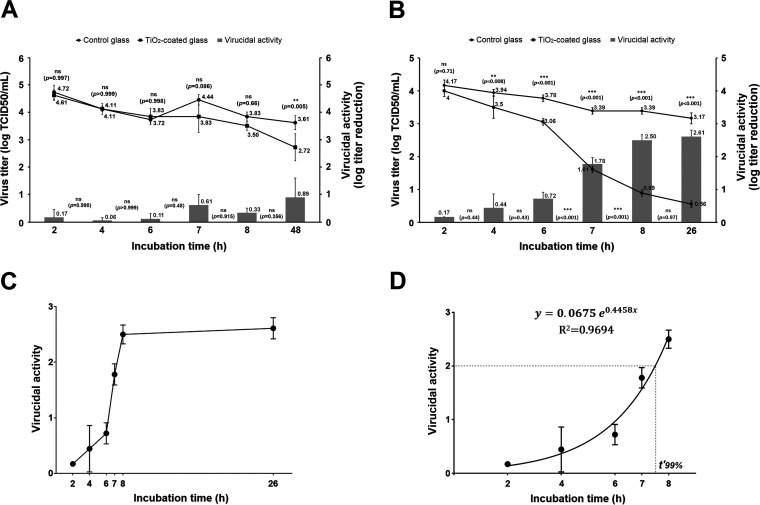
Virucidal profile of TiO_2_-coated glass against GFP-expressing human coronavirus 229E. Time course survival of HCoV-229E/GFP on TiO_2_-coated glass and uncoated float glass when the experiment was performed in the absence of light (A) or when the glass samples were preactivated with UV-A light and further incubated with viruses in the presence of D65 light throughout the experiment (B). Virus titers (lines [left axis]) recovered from both uncoated control float glass (●) and TiO_2_-coated glass (▪) surfaces were compared at each time point using Sidak’s multiple mean comparison test (*P* values shown above the lines). The virucidal indexes between time points (bars [right axis]) were compared using the Tukey’s multiple mean comparison test. The *P* values shown refer to statistical comparison between adjacent virucidal values (see details in the text). (C) Graph of virucidal activity versus elapsed time, showing the kinetics of HCoV-229E/GFP virus inactivation on TiO_2_-coated surfaces. (D) Exponential phase of virus inactivation on TiO_2_-coated surface (see details in the text). Data are presented as means plus standard deviations.

Our data indicate that the absence of light throughout the experiment prevented the activation of the virucidal mechanism of TiO_2_ in the commercial coated glass. The virus infectious titers recovered from both the coated and the control uncoated glass were similar during the time course of the experiment (Sidak’s multiple-comparison test, *P > *0.05), except for the last time point, 48 h (*P = *0.005) (Fig. 1A). The incubation times after which the initial titer dropped to half (*t*_50%_, or half-life) were 11.1 h and 16.0 h for the commercial TiO_2_-coated glass and control float glass, respectively (Table 1). Incubation for 36.8 h and 53.6 h on the coated glass and control glass, respectively, reduced the virus titer by 1 log, equivalent to 90% inactivation of viral particles (time to 90% inactivation [*t*_90%_]). Further incubations for 73.8 h and 106.4 h are theoretically required to achieve 99% virus inactivation on both glasses, respectively (*t*_99%_) (Table 1).

**TABLE 1:** Survival rates of HCoV-229E/GFP on glass surfaces

Illumination	Sample	*t*_50%_ (half-life), h	*t*_90%_ (1 log), h	*t*_99%_ (2 log), h
No light	Control	16.0	53.2	106.4
	TiO_2_-coated glass	11.1	36.9	73.80
UV-A+D65	Control	8.78	29.2	58.3
	TiO_2_-coated glass	0.59	1.97	3.94

Although these titer decay rates appear slightly different between both surfaces, the values found are consistent with the variable inactivation rates previously reported for HCoV-229E and other coronaviruses. For example, Chin et al. reported a SARS-CoV-2 half-life on regular glass in the range of 1.2 to 4.8 h, and a *t*_99%_ from 48 to 96 h (10). Behzadinasab et al. were able to recover viable SARS-CoV-2 after 24 h incubation on uncoated glass, the *t*_99%_ being thus an undetermined value beyond this time (11). Although there is little information on the precise HCoV-229E half-life and its *t*_90%_ and *t*_99%_ values in the literature, no viable HCoV-229E was recovered after incubation on glass surfaces for 5 days (12) or 7 days (13).

Bonil et al. reported a half-life of 6.9 h and a *t*_90%_ of 21.6 h for SARS-CoV-2 on classic glass and acrylic glass (14). These researchers found the same values for photocatalytic TiO_2_-coated glass in the absence of light, endorsing our finding that this commercial coated glass behaves as does regular glass when no illumination is used. Since HCoV-229E/GFP titers recovered from both the coated glass and bare glass after 2- to 8-h incubations were similar (Fig. 1A, lines), no virucidal activity can be attributed to the coating material present on the former. The virucidal indexes found throughout the time course of the experiment in the absence of light were in the range of 0.06 to 0.89 (Tukey’s multiple-comparison test, *P > *0.05) (Fig. 1A, bars), far from the 2-log reduction required to reach a 99% virion inactivation. The significant difference in virus titers found for 48 h of incubation may be due to intrinsic virucidal activity of TiO_2_ even in the absence of photoactivation, as was recently reported by Khaiboullina et al. (15).

### D65 illumination triggers photocatalytic activation of TiO_2_-coated glass virucidal activity, regardless of UV-A light preactivation.

HCoV-229E/GFP virus showed a different stability pattern on TiO_2_-coated glass when this surface was preactivated and further maintained under daylight (D65) illumination throughout the virucidal assay. The virus decay on control float glass under these conditions was of note and consistent with that observed during the virucidal experiment in the absence of light, with a half-life of 8.78 h and a *t*_90%_ and a *t*_99%_ of 29.2 h and 58.3 h, respectively (Table 1). Conversely, HCoV-229E/GFP virus infectivity dramatically decayed on D65-irradiated coated glass, with a half-life of 0.59 h, a *t*_90%_ of 1.97 h, and a *t*_99%_ of 3.94 h (Table 1), suggesting a direct role for this kind of illuminant on the photocatalytic activation of virucidal components present in the coating. The surrogate model HCoV-229E/GFP on D65-activated TiO_2_-coated glass performed differently from SARS-CoV-2 on photocatalytically activated TiO_2_ glass from another glass supplier (14), as the latter showed a 4.1-h half-life and a *t*_90%_ of 13.5 h. However, it should be mentioned that not only were the virus species tested by these authors different, but also, the type of TiO_2_ coating and deposition method varied, thus explaining the different survival rates (14).

As shown in Fig. 1B (lines), statistical differences between virus titers recovered from the TiO_2_-coated glass and those from control glass became significant at 4 h incubation (Sidak’s test, *P = *0.008) and continued to increase up to 26 h incubation (Sidak’s test, *P < *0.001). The virucidal activity attributed to the coating itself was calculated for each incubation time as the difference between the log titer of virus recovered from the control glass and that of the coated glass from the same time point. As the titers from the coated glass prominently dropped with incubation time, the virucidal activity indexes of these samples increased concomitantly, with statistically significant differences appearing at 6 h incubation (Tukey’s test, *P < *0.001) (Fig. 1B, bars).

Interestingly, the virucidal activity of TiO_2_ coating against HCoV-229E/GFP fitted a biphasic kinetic model, with a well-defined exponential phase from the 2-h to the 8-h incubation time point and a stationary phase beyond 8 h, where no further significant decrease of virus titer was achieved (Tukey’s test for 8 h versus 26 h, *P = *0.11), as can be seen in the expanded elapsed-time graph shown in Fig. 1C. Taking the exponential phase of this virucidal curve, we calculated a normalized time to 99% inactivation (*t*′_99%_) corresponding to 7.6 h (Fig. 1D). This value is larger than and should not be confounded with the prior *t*_99%_ value, as it refers to the virucidal effect of the coating itself, excluding other factors contributing to virus decay (that also occur on the bare float glass), such as exposure to any surface over time.

Recently, biphasic inactivation kinetics has also been observed for the human coronavirus SARS-CoV-2 decay on smooth (nonporous) surfaces such as glass, stainless steel, and plastic (10). The authors of this work described the occurrence of two separate, well-defined half-lives of SARS-CoV-2 on these surfaces: a first “short half-life,” calculated from the initial phase of the curve (with a more pronounced slope), which takes place approximately between 0 and 6 h of incubation, and a second “long half-life,” starting beyond 8 to 10 h of incubation and continuing to 20 to 50 h depending on the material, which is calculated within the plateau or stationary region of the decay curve, as it slowly approaches the zero value of virus titer (10). Our findings on the virucidal activity of D65 light-irradiated TiO_2_-coated glass against human coronavirus 229E/GFP are consistent with this kind of kinetic behavior, as discussed above (Fig. 1C). Whether this effect is unique to coronavirus inactivation or can also be observed for other viruses remains unknown and merits further investigation.

**FIG 2:**
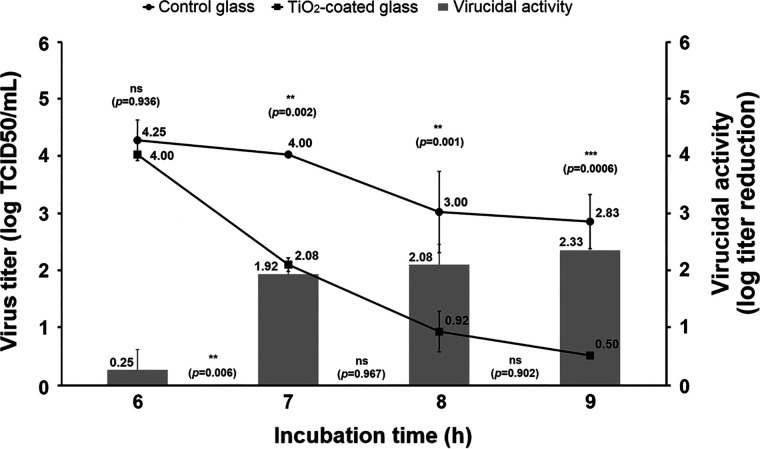
Virucidal performance of TiO_2_-coated glass against GFP-expressing human coronavirus 229E when illuminated with D65 light throughout the incubation with virus, in the absence of previous activation with UV-A light. This experiment was performed in duplicate. Virus titers (lines [left axis]) recovered from both uncoated control float glass (●) and TiO_2_-coated (▪) surfaces were compared at each time point using Sidak’s multiple mean comparison test (*P* values shown above the lines). The virucidal indexes between time points (bars [right axis]) were compared using the Tukey’s multiple mean comparison test. The *P* values shown refer to statistical comparison between adjacent virucidal values (see details in the text).

Despite being considered the most efficient photocatalytic material and the most effective antimicrobial of its class, some drawbacks have been reported for TiO_2_ that supposedly limit its practical application. For instance, the relatively low band gap of its anatase phase (3.2 eV) implies that it can be photoactivated only with light with a wavelength of <387.5 nm (approximately 3% of the solar light spectrum) (16). For this reason, our first virucidal approaches using TiO_2_-coated glass were developed following preactivation of the test material with UV-A light. Surprisingly, a virus-inactivating capacity similar to that initially described was observed when the virucidal assay took place solely with D65 light, without UV-A preactivation of the glass. This experiment was carried out with selected time points from the virucidal assay described in Materials and Methods (“Test glass and virucidal assays”), i.e., 6, 7, 8, and 9 h incubation. The virucidal indexes ranged from 0.25 to 2.83 log virus titer reduction (Fig. 2). These values are similar to those obtained when the test glass plates were preactivated with UV-A light, which ranged from 0.72 to 2.5 in a similar time-lapse experiment (Fig. 1B), suggesting that D65 light irradiation is necessary and sufficient to trigger photocatalytically activated virucidal activity of TiO_2_-coated glass. Despite its being generally accepted that TiO_2_ is photoactivated in the UV region of the spectrum, several studies have shown that virucidal activity is also achieved under visible light against several enveloped viruses, such as herpesviruses (17), hepatitis B virus (18) and influenza viruses (19). A possible explanation could be the presence of other chemical elements on the glass that might contribute to the extended wavelength of TiO_2_ photoactivation, as has previously been reported (20). Whether other types of illuminants also can photoactivate the virucidal properties of this material remains unknown and merits further investigation.

### Viral RNA load recovered from test surfaces does not correlate with virus infectious titers.

**FIG 3:**
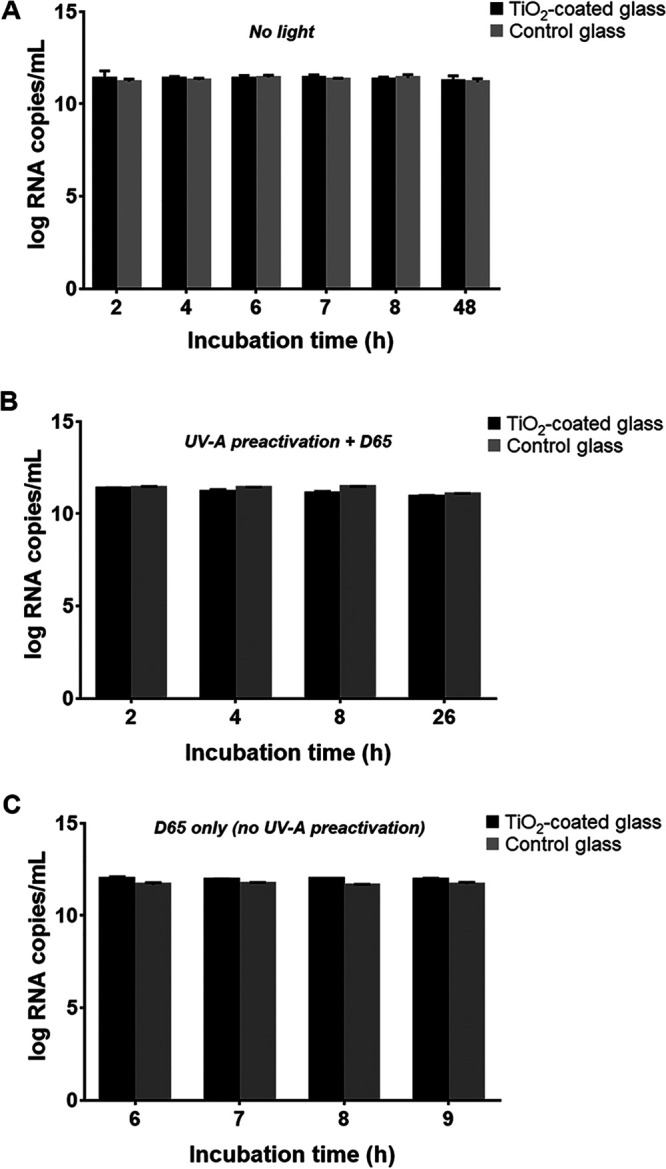
Quantification of viral genomic RNA detected in virus preparations recovered from the virucidal assays described in Materials and Methods (“Test glass and virucidal assays”), performed under different illumination conditions. (A) Amounts of viral RNA recovered after incubation of virus on TiO_2_-coated and float glasses in the absence of light; (B) amounts of virus RNA detected in virus recovered from the test glasses subjected to preactivation with UV-A light and further incubation with D65 light; (C) amounts of virus RNA from samples recovered from the test glasses in the presence of D65 light without UV-A preactivation. The viral genomic RNA in the samples was quantified using RT-qPCR by extrapolation from the standard curve (Fig. S2), run in triplicate together with the rest of samples (see details in the text).

In an attempt to ascertain whether coronavirus exposure to TiO_2_ coating has an effect on the recovery of its genomic RNA after virucidal assays, the viruses washed off from both the coated and the control glass (see Materials and Methods, “Test glass and virucidal assays”) were subjected to RNA extraction and quantification by reverse transcription-quantitative PCR (RT-qPCR). As shown in Fig. 3, the virus RNA copy numbers remained steady throughout the time course of the experiments for all assayed illumination conditions (*P > *0.05, two-way ANOVA or Kolmogorov-Smirnov nonparametric test), and they were also similar among control glass- and TiO_2_ glass-exposed viruses for all assay conditions and incubation time (*P > *0.05, two-way analysis of variance [ANOVA] or Wilcoxon matched-pairs signed ranked nonparametric test). The virus RNA quantity ranged from 8.31 × 10^10^ to 1.13 × 10^12^ copies per mL. This result was expected, because the loss of the integrity of viral envelope that ultimately leads to a drop in infectious titer has little or no effect on the amount of total viral RNA present in the sample, which can easily be detected via a sensitive technique such as RT-qPCR. In other words, the amount of virus genomic RNA in treated samples is not a good predictor of virucidal activity, as viral RNA could have been released from disrupted virions. In fact, the RNA might even be heavily damaged due to TiO_2_-induced oxidative stress and still be detected, as the RT-qPCR used in this study specifically targets a very short sequence within the virus genome. Our findings agree with previous data on SARS-CoV-2 RNA detection upon incubation with different materials (14). In that report, the authors used an inoculum of 1,000-fold less infectious virus (compared to the present study) and incubated for 0 to 24 h. The viral RNA quantities remained stable around 10^8^ copies/mL for all studied materials, including TiO_2_-coated glass, even when the infectivity of the same samples had drastically dropped by 2 log (14).

### Human coronavirus 229E incubated on TiO_2_-coated glass with D65 illumination showed altered morphologies under electron microscopy.

**FIG 4:**
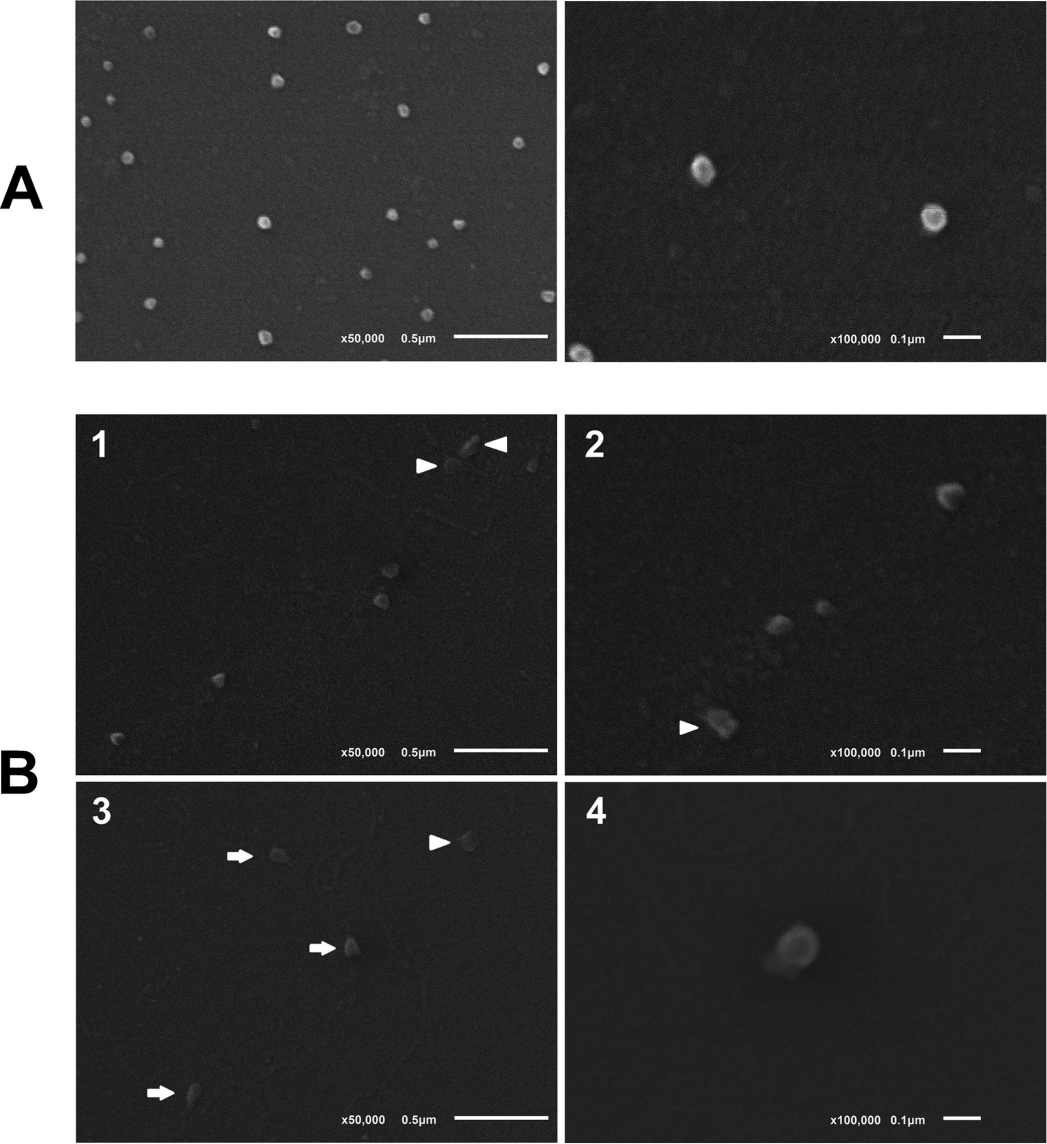
Scanning electron microscopy (SEM) images of gold sputter-coated control float glass (A) and TiO_2_-coated glass (B), after 20 h incubation each with 5 × 10^4^ TCID_50_ of HCoV-229E/GFP in the presence of D65 light. Magnifications, ×50,000 (left panels) and ×100,000 (right panels). See further details in the text.

Scanning electron microscopy (SEM) analyses of human coronavirus 229E/GFP incubated on float glass showed numerous virions of around 70 to 100 nm, when observed at a magnification of ×50,000 (Fig. 4A, left). Although coronaviruses can exhibit some degree of virion pleomorphism, the viral particles on float glass looked generally spherical with regular edges and showed very little variation in morphology and size, which is indicative of viable, potentially infectious virus. A detailed observation at ×100,000 revealed that virions appeared as hypo-electron-dense circular structures surrounded by a hyper-dense halo (Fig. 4A, right), as previously reported for most members of the family *Coronaviridae* (21).

Conversely, the incubation of virus on the TiO_2_-coated glass and further illumination with D65 light for 20 h led to a decrease in the number of total viral particles that were observed under SEM (Fig. 4B, panels 1 and 3), as well as a plethora of morphological alterations (Fig. 4B, panels 1 to 4) that rendered Bioclean-incubated virions clearly distinguishable from viruses exposed to float glass. Such alterations included the loss of part of the electron-dense (bright) material that surrounds the normal virion, compatible with the physical damage of virion surface proteins (e.g., S, M, and E proteins), the loss of spherical morphology, and the appearance of fusiform shapes (Fig. 4B, arrows). The presence of virion debris or heavily disrupted particles, reminiscent of virion ghosts, was also noted (Fig. 4B, arrowheads). The deformation of virions may be related to water intake following ROS-induced viral envelope instability, as the glass becomes more hydrophilic due to TiO_2_-induced wettability, and the particle size can eventually reach a critical threshold beyond which the virion bursts, releasing the nucleocapsid and other internal components. We believe that this is what was micrographed in Fig. 4B, panel 4. The image shows a viral particle slightly larger than usual (104-nm diameter) that appears partially disrupted on its bottom left flank, with part of its content displayed on the outside.

**FIG 5:**
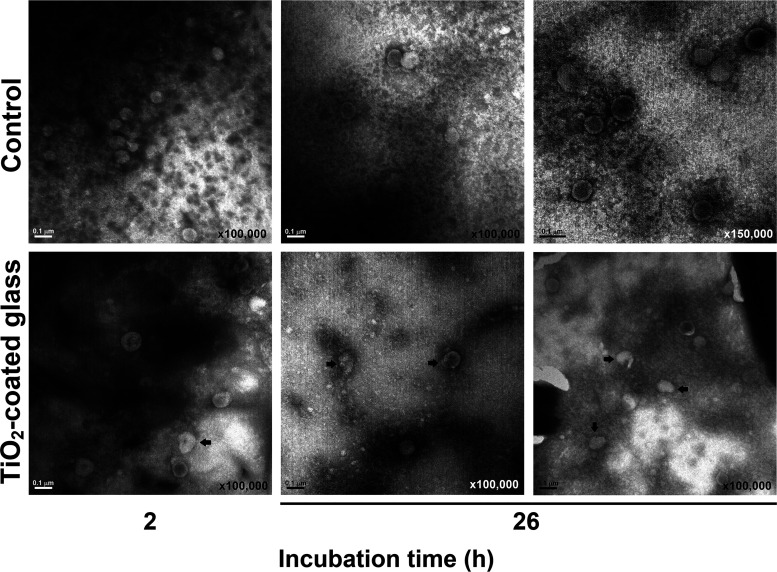
Transmission electron microscopy (TEM) images of negatively stained HCoV-229E/GFP recovered from float glass and TiO_2_-coated glass, after 2 and 26 h exposure. Two different fields are shown for each sample at the 26-h time point. Bars, 0.1 μm.

Transmission electron microscopy (TEM) images of viruses recovered from 2- and 26-h incubation time points (see Materials and Methods, “Test glass and virucidal assays”) (Fig. 1B) showed a similar pattern. Figure 5 shows the morphological features of virions incubated on control float glass and TiO_2_-coated glass, for 2 and 26 h. While most virions on the control glass displayed a round, regular shape at both incubation times, the number of morphologically altered and disrupted virions (arrows) increased between 2 and 26 h incubation for the TiO_2_-exposed viruses (Fig. 5).

**FIG 6:**
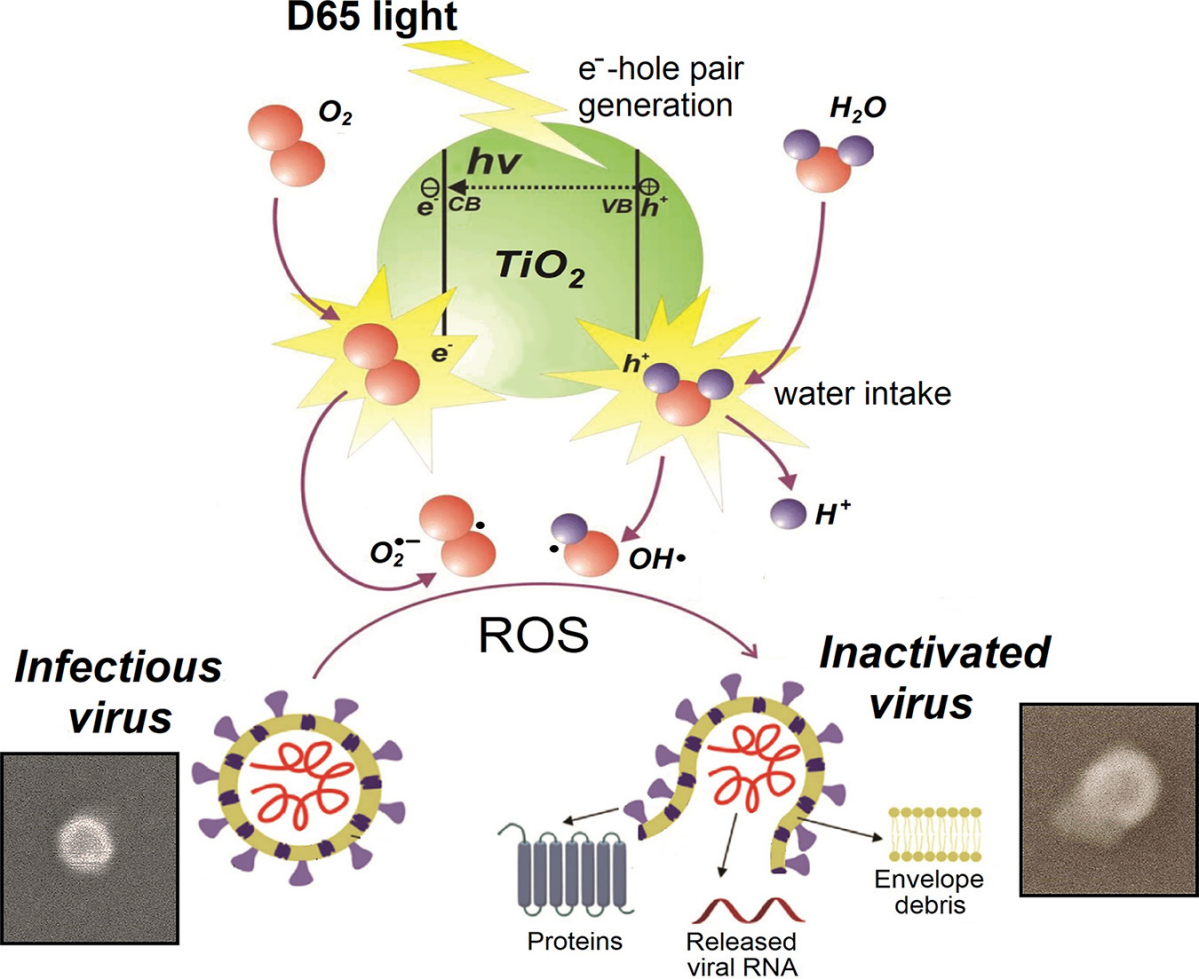
Schematic model for photocatalysis-triggered virucidal activity of TiO_2_ against coronaviruses. Upon excitation by light, the photon energy (*hv*) generates an electron-hole pair on the TiO_2_ surface, with holes located in the valence band (VB) of the semiconductor and electrons accumulating in the conduction band (CB). An electron-hole pair is a highly unstable state with strong oxidation/reduction power that converts water and oxygen into hydroxyl radicals (^·^OH), superoxide ions (O_2_^·−^) and hydrogen peroxide (H_2_O_2_) in the vicinity of holes within the valence band. These reactive oxygen species (ROS) exert their virucidal action through oxidative stress, causing virus genome damage, lipid peroxidation with the consequent disruption of the viral envelope, virion osmotic shock, and lysis, which is ultimately associated with the release of the nucleocapsid and other virion components and the loss of viral infectivity (virus inactivation).

It has been well established that, because of the hydrophobic nature of the viral envelope, there is a direct correlation between the wettability (hydrophilic nature) of a particular material and its inactivating capacity against enveloped viruses. For example, the virus persists longer on hydrophobic materials such as polypropylene than on stainless steel (moderately hydrophilic), and it is also more stable on stainless steel and plastic than on glass and cotton/cellulose-based materials, which are considerably hydrophilic (8). Furthermore, the TiO_2_ coating confers additional hydrophilicity to the glass upon chemical transformation during daylight irradiation. TiO_2_ coatings are capable of trapping water molecules after being irradiated with visible light in a time-dependent manner, thus becoming increasingly hydrophilic (16), which could explain the enhanced virus inactivation achieved on this surface over time, in the presence of D65 light. Although the precise antimicrobial mechanisms of photocatalytic materials such as TiO_2_ are still under debate, their microbicidal properties have been generally attributed to the oxidative stress resulting from ROS generation. These ROS include free superoxide and hydroxyl radicals that react with microbe membrane components oxidizing them and ultimately disrupting the membrane itself, with the concurrent cell lysis. Another putative mechanism involves the direct damage of the microbe’s genetic material by ROS. In the case of enveloped viruses such as coronaviruses, because of the lipidic nature of the viral envelope, these ROS most likely act similarly, by disrupting the viral envelope, leading to virion lysis with the consequent release of the nucleocapsid and other virion components and the ultimate loss of virus infectivity (Fig. 6).

### Conclusions.

One of the best approaches to control the COVID-19 pandemic is to reduce virus transmission. Surface contamination has been acknowledged as an important contributor to SARS-CoV-2 spread. Therefore, in addition to antiviral drugs and vaccines, the use of personal protective elements and physical containment barriers (such as masks, gloves, or indoor separating glass walls) helps increase safety within closed environments and have proven invaluable in preventing contagion. Redesigning or engineering these barriers so that the virus cannot remain infectious could make a difference in SARS-CoV-2 and related pathogen epidemiology. The introduction of additives or coatings with virucidal activity could potentiate the protective effects of these safety elements to serve not only as physical barriers but also as virus “killers” to work upon contaminated hand touch- or aerosol-mediated surfaces. The virucidal properties found for this commercial TiO_2_-coated glass are one example of a material application with a potential epidemiologic impact. To the best of our knowledge, this is the first report showing direct visual evidence of human coronaviruses being damaged and morphologically altered following exposure to this photocatalyst, under scanning and transmission electron microscopes. Compared to regular disinfection agents, such as chlorine, alcohol, etc., the TiO_2_-based antiviral approaches have several advantages. First, the lack of toxicity of TiO_2_ makes it suitable for use in a wide range of environments and applications without compromising human health; second, as a catalyst, the material is not consumed in the reaction itself, which allows the coatings to function indefinitely. Where daylight-like illumination is present, such photocatalytic coatings could be incorporated onto daily-life high-touch surfaces, such as screens and urban furniture (furniture in public settings), thus giving them an added value not only in decelerating spread of the current pandemic but also in preventing future outbreaks.

## MATERIALS AND METHODS

### Virus and cells.

A recombinant human coronavirus 229E expressing the green fluorescent protein (HCoV-229E/GFP), constructed in the laboratory of Volker Thiel (University of Bern, Switzerland) (22) was kindly donated by Luis Enjuanes (Centro Nacional de Biotecnología, CSIC, Madrid, Spain), with permission. The virus was propagated in the human hepatoma cell line HuH-7 (23), a gift from Luis Enjuanes’s laboratory. HuH-7 cells were grown in Dulbecco’s modified minimal essential medium (DMEM) supplemented with 10% heat-inactivated fetal bovine serum (Gibco), MEM nonessential amino acids (Thermo Fisher Scientific), 0.2 M GlutaMAX (Thermo Fisher Scientific) and antibiotic-antimycotic solution (Thermo Fisher Scientific). Confluent or near-confluent HuH-7 cell monolayers were maintained under similar culture conditions, with the serum concentration reduced to 2%. All the experimental procedures performed in this research complied with the good laboratory practice recommendations for a biosafety level 2 virology facility.

### Virus stock production and titration.

HCoV-229E/GFP high-titer virus stocks were produced by ultracentrifugation through a sucrose cushion as previously described for other enveloped viruses (24), with minor modifications. Briefly, near-confluent HuH-7 cell monolayers grown in T150 flasks were infected at a multiplicity of infection (MOI) of 0.1 and incubated at 37°C in a 5% CO_2_ atmosphere incubator, for 60 to 72 h. After this incubation period, the free and cell-associated viruses were harvested together by scraping the monolayers into the culture medium. The preparations were frozen and thawed three times and clarified by centrifugation at 3,000 × *g* for 15 min in a Beckman Coulter Allegra X-22R centrifuge. The resulting supernatants were filtered through 0.45-μm polyvinylidene difluoride (PVDF) membranes and further centrifuged through a 30% sucrose 4-mL cushion, at 25,000 rpm in a Kontron Centrikon T-1180 ultracentrifuge (TST-28.38 rotor) for 2.5 h (4°C). Each virus pellet produced from two T150 flasks was resuspended in 200 μL Dulbecco modified phosphate-buffered saline (d-PBS).

Virus stock titrations were performed by the endpoint dilution method described by Reed and Muench (25) and the viral titers were expressed as mean infectious doses (TCID_50_)/mL. For the TCID_50_ assays, the appearance of at least one green-fluorescent (GFP-expressing) cell using the Eclipse Ts2-FL fluorescence microscope (Nikon) was considered indicative of a positive (infected) well, rather than the sole observation of virus-associated cytopathic effect. This fluorescence-based method allowed a more objective and accurate virus quantitation.

### Test glass and virucidal assays.

TiO_2_-coated glass (Bioclean; test glass) and bare glass (float glass, control) (5 cm by 5 cm by 0.35 cm) were obtained from Saint-Gobain Building Glass and were preactivated by UV-A light exposure for 4 h, using a QUV accelerated weathering tester (Q-lab). After preactivation, each glass specimen was placed inside a sterile 10-cm-diameter petri dish. The virucidal experiments were carried out according to the general guidelines of ISO standard 21702:2019 (26), with minor modifications. Briefly, 10^6^ TCID_50_ of HCoV-229E/GFP stock was deposited dropwise on each test surface and covered with a 4- by 4-cm piece of polyvinyl chloride (PVC) transparent film. The resulting assemblies were incubated at room temperature for 2, 4, 6, 7, 8, 9, 12, or 26 h, under illumination conditions that simulate daylight (D65). D65 illumination was generated with the aid of a CMlite model GLE-M5/17 lamp (GTI Graphic Technology, Inc.), placed inside the biosafety class IIB airflow cabinet. The light source was 25 cm away, above the glass (estimated maximum irradiance = 0.18 mW/cm^2^). After the indicated incubation times, the PVC film was separated with sterile tweezers, and the viruses from both surfaces were recovered by thorough washing with DMEM supplemented with 2% fetal bovine serum (the washing step resulted in a 1/10 dilution of the initially deposited virus). A similar procedure was done in the presence of D65 light in which the glass specimens were not subjected to UV-A preactivation. A control experiment in the absence of any type of light was also performed.

The residual infectivity of recovered viral particles was determined using the endpoint titration method. For this purpose, serial 10-fold dilutions from the samples were made and used to inoculate HuH-7 confluent monolayers in 96-well plates, with 100 μL/well using 6 wells per dilution (sextuplicate). After 72 h incubation (37°C, 5% CO_2_), the monolayers were observed under the fluorescence microscope, and the number of wells per dilution showing at least one green-fluorescent (infected) cell was recorded. The dilution infecting 50% of wells (TCID_50_) was calculated as described by Reed and Muench (25), and the infectious titer of the original sample was expressed as TCID_50_/mL. In order to quantify the virucidal activity of the test glass, the whole experiment was performed in parallel with a regular glass lacking the TiO_2_ coating (control glass). A virucidal index for each time point was calculated by subtracting the logarithm of virus titer found in the test glass from the logarithm of virus titer recovered from the paired control glass.

### RNA extraction and RT-qPCR.

The RNA from viruses recovered after time course incubations on both test surfaces (see “Test glass and virucidal assays” above) was extracted with the RNeasy minikit (Qiagen) and quantified by RT-qPCR using the TaqMan Virus Fast one-step master mix (Applied Biosystems) in a QuantStudio 5 real-time PCR thermocycler (Applied Biosystems). For this purpose, the following oligonucleotides were designed with the aid of Primer Express 3.0.1 software (Applied Biosystems): 5′-GTCGCAGTGCCATAGAAGACATAC-3′ (forward primer), 5′-GTCAGCAATGGAAAGACCCTTAGT-3′ (reverse primer), and 5′-TAGCAAACTTGTTACTTCTGGACTTGGCACTGTG-3′ (TaqMan probe labeled with 5′ ABY dye and the 3′ nonfluorescent proprietary quencher QSY) (Thermo Fisher Scientific). The primers and probe were based on the human coronavirus 229E reference sequence in GenBank (accession number NC_002645) and were used at 450 nM and 125 nM, respectively. The ROX (carboxyrhodamine) dye present in the master mix was used as the passive reference, and the cycling conditions were those recommended by the TaqMan master mix’s manufacturer. For absolute (copy number) quantification of samples, a standard curve was generated (Fig. S2) by amplifying (in triplicate) serial decimal dilutions of HCoV-229E/GFP RNA isolated from purified virions and quantifying with the aid of a Qubit 2.0 fluorometer (Invitrogen).

### Electron microscopy analyses.

For scanning electron microscopy (SEM) analyses, 5 × 10^4^ TCID_50_ of HCoV-229E/GFP were evenly deposited on each surface (TiO_2_-coated glass and bare float glass) and incubated in the presence of D65 light for 20 h. Afterwards, both surfaces were sputter coated with gold by using a Balzer SCD004 sputter coater and examined through a JEOL model JSM-6610LV scanning electron microscope, with a 20-kV acceleration voltage and a working distance of 10 mm.

The biological specimens for transmission electron microscopy (TEM) were prepared as follows: 20-μL drops of virus recovered from control and TiO_2_-coated glass, after the virucidal experiment (time points 2 and 26 h; see “Test glass and virucidal assays”), were deposited on a petri dish. A Formvar-copper 200 mesh grid (Electron Microscopy Sciences; model FF200-CU, catalog no. 215-412-8400) was floated on each sample drop, Formvar side down, for 10 min. Afterwards, the grids were blotted on their sides on filter paper to remove the liquid excess and were further floated (also Formvar side down) for 1 min on a 4% uranyl acetate solution. The grids were blotted again, allowed to dry overnight, and imaged using a JEOL model JEM-1011 electron microscope. Samples were micrographed at ×100,000 and ×150,000 magnifications, with an acceleration voltage of 100 kV.

### Statistical analyses.

For each glass material, parameters such as intrinsic virus half-life (*t*_50%_) and time to reduce titers by 1 log unit (*t*_90%_) and 2 log units (*t*_99%_) were calculated by using regression analyses from the time course curves generated from data. The experiments were performed in triplicate, and virus titers (log TCID_50_/mL), virucidal indexes, and RNA copy numbers collected across time points were assessed for normality and homoscedasticity using the Shapiro-Wilk and the Levene tests, respectively. *P* values of >0.05 were considered indicative of data normality and variance homogeneity. Multiple mean comparison analyses were performed with ANOVA. Paired-means comparisons were performed using Student's *t* test, unless otherwise indicated. *P* values of <0.05 were considered indicative of significant differences among means. The different means were assessed using Sidak’s or Tukey’s *post hoc* multiple-comparison tests. All experiments were performed in triplicate, and data are presented as means and standard deviations. All statistical analyses were done using GraphPad Prism v7 software.

### Data availability.

The data sets generated in this study can be found in the paper.

## References

[1] WHO. 2022. COVID-19 dashboard. https://covid19.who.int/. Accessed 7 March 2022.

[2] WHO. 2022. COVID-19: landscape of novel coronavirus candidate vaccine development worldwide. https://www.who.int/publications/m/item/draft-landscape-of-covid-19-candidate-vaccines. Accessed 7 March 2022.

[3] Fernstrom A, Goldblatt M. 2013. Aerobiology and its role in the transmission of infectious diseases. J Pathog 2013:493960. doi:10.1155/2013/493960. 23365758 PMC3556854

[4] Boone SA, Gerba CP. 2007. Significance of fomites in the spread of respiratory and enteric viral disease. Appl Environ Microbiol 73:1687–1696. doi:10.1128/AEM.02051-06. 17220247 PMC1828811

[5] Cai J, Sun W, Huang J, Gamber M, Wu J, He G. 2020. Indirect virus transmission in cluster of COVID-19 cases, Wenzhou, China, 2020. Emerg Infect Dis 26:1343–1345. doi:10.3201/eid2606.200412. 32163030 PMC7258486

[6] Foster HA, Ditta IB, Varghese S, Steele A. 2011. Photocatalytic disinfection using titanium dioxide: spectrum and mechanism of antimicrobial activity. Appl Microbiol Biotechnol 90:1847–1868. doi:10.1007/s00253-011-3213-7. 21523480 PMC7079867

[7] Markowska-Szczupak A, Ulfig K, Morawski AW. 2011. The application of titanium dioxide for deactivation of bioparticulates: an overview. Catalysis Today 169:249–257. doi:10.1016/j.cattod.2010.11.055.

[8] Bueckert M, Gupta R, Gupta A, Garg M, Mazumder A. 2020. Infectivity of SARS-CoV-2 and other coronaviruses on dry surfaces: potential for indirect transmission. Materials (Basel) 13:5211. doi:10.3390/ma13225211. 33218120 PMC7698891

[9] Butot S, Baert L, Zuber S. 2021. Assessment of antiviral coatings for high-touch surfaces by using human coronaviruses HCoV-229E and SARS-CoV-2. Appl Environ Microbiol 87:e0109821. doi:10.1128/AEM.01098-21. 34288707 PMC8432523

[10] Chin AWH, Chu JTS, Perera MRA, Hui KPY, Yen HL, Chan MCW, Peiris M, Poon LLM. 2020. Stability of SARS-CoV-2 in different environmental conditions. Lancet Microbe 1:e10. doi:10.1016/S2666-5247(20)30003-3. 32835322 PMC7214863

[11] Behzadinasab S, Chin A, Hosseini M, Poon L, Ducker WA. 2020. A surface coating that rapidly inactivates SARS-CoV-2. ACS Appl Mater Interfaces 12:34723–34727. doi:10.1021/acsami.0c11425. 32657566 PMC7385996

[12] Warnes SL, Little ZR, Keevil CW. 2015. Human coronavirus 229E remains infectious on common touch surface materials. mBio 6:e01697-15. doi:10.1128/mBio.01697-15. 26556276 PMC4659470

[13] Bonny TS, Yezli S, Lednicky JA. 2018. Isolation and identification of human coronavirus 229E from frequently touched environmental surfaces of a university classroom that is cleaned daily. Am J Infect Control 46:105–107. doi:10.1016/j.ajic.2017.07.014. 28893443 PMC7115338

[14] Bonil L, Lingas G, Coupeau D, Lucet J-C, Guedj J, Visseaux B, Muylkens B. 2021. Survival of SARS-CoV-2 on non-porous materials in an experimental setting representative of fomites. Coatings 11:371. doi:10.3390/coatings11040371.

[15] Khaiboullina S, Uppal T, Dhabarde N, Subramanian VR, Verma SC. 2020. Inactivation of human coronavirus by titania nanoparticle coatings and UVC radiation: throwing light on SARS-CoV-2. Viruses 13:19. doi:10.3390/v13010019. 33374195 PMC7824386

[16] Ratova M, Redfern J, Verran J, Kelly PJ. 2018. Highly efficient photocatalytic bismuth oxide coatings and their antimicrobial properties under visible light irradiation. Appl Catal B Environ 239:223–232. doi:10.1016/j.apcatb.2018.08.020.

[17] Hajkova P, Spatenka P, Horsky J, Horska I, Kolouch A. 2007. Photocatalytic effect of TiO2 films on viruses and bacteria. Plasma Process Polym 4:S397–S401. doi:10.1002/ppap.200731007.

[18] Xu R, Liu X, Zhang P, Ma H, Liu G, Xia Z. 2007. The photodestruction of virus in nano-TiO2 suspension. J Wuhan Univ Technol 22:422–425. doi:10.1007/s11595-006-3422-6.

[19] Guillard C, Bui TH, Felix C, Moules V, Lina B, Lejeune P. 2008. Microbiological disinfection of water and air by photocatalysis. C R Chim 11:107–113. doi:10.1016/j.crci.2007.06.007. 32288747 PMC7110965

[20] Fujishima A, Zhang X. 2006. Titanium dioxide photocatalysis: present situation and future approaches. C R Chim 9:750–760. doi:10.1016/j.crci.2005.02.055.

[21] Haddad G, Bellali S, Fontanini A, Francis R, La Scola B, Levasseur A, Bou Khalil J, Raoult D. 2020. Rapid scanning electron microscopy detection and sequencing of severe acute respiratory syndrome coronavirus 2 and other respiratory viruses. Front Microbiol 11:596180. doi:10.3389/fmicb.2020.596180. 33329483 PMC7711091

[22] Cervantes-Barragan L, Zust R, Maier R, Sierro S, Janda J, Levy F, Speiser D, Romero P, Rohrlich PS, Ludewig B, Thiel V. 2010. Dendritic cell-specific antigen delivery by coronavirus vaccine vectors induces long-lasting protective antiviral and antitumor immunity. mBio 1:e00171-10. doi:10.1128/mBio.00171-10. 20844609 PMC2939679

[23] Nakabayashi H, Taketa K, Miyano K, Yamane T, Sato J. 1982. Growth of human hepatoma cells lines with differentiated functions in chemically defined medium. Cancer Res 42:3858–3863. 6286115

[24] Kutner RH, Zhang XY, Reiser J. 2009. Production, concentration and titration of pseudotyped HIV-1-based lentiviral vectors. Nat Protoc 4:495–505. doi:10.1038/nprot.2009.22. 19300443

[25] Reed LJ, Muench H. 1938. A simple method of estimating fifty per cent endpoints. Am J Hyg 27:493–497. doi:10.1093/oxfordjournals.aje.a118408.

[26] ISO. 2019. Measurement of antiviral activity on plastics and other non-porous surfaces. https://www.iso.org/standard/71365.html. Accessed 10 July 2021.

