# Supplementary material for: Virucidal Properties of Photocatalytic Coating on Glass against a Model Human Coronavirus
Source: Microbiol Spectr. 2022 May 4;10(3):e00269-22. doi: 10.1128/spectrum.00269-22 (PMC9241891; doi:10.1128/spectrum.00269-22)

FIGURE S1: (A) Photograph showing the experimental setup of Cmlite lamp inside the biosafety cabinet, elevated 25 cm above the Petri dishes containing the test glass surfaces. The lower panels show the test glass-containing Petri dishes with the virus preparation deposited dropwise, before (left) and after (right) covering with the PVC film. (B) Microphotograph of HuH-7 cells monolayers inoculated with HCoV-229E/GFP virus recovered from the glass surface, showing the criteria for negativity (left panels) and positivity (right panels) during virucidal assay titrations. The upper panels show the aspect of negative and positive wells using trans-mitted bright light. The wells displaying virus-induced cytopathic effects (upper right panel) are clearly distinguishable from uninfected wells (upper left panel). The CPE consisted of cell rounding, refringence and loss of confluency and monolayer integrity. The lower panels show the same fields under epifluorescence illumination using the green filter. The appearance of at least one green cell was considered indicative of virus infection and led to the unambiguous classification of a well as positive (bars length=400 µm).

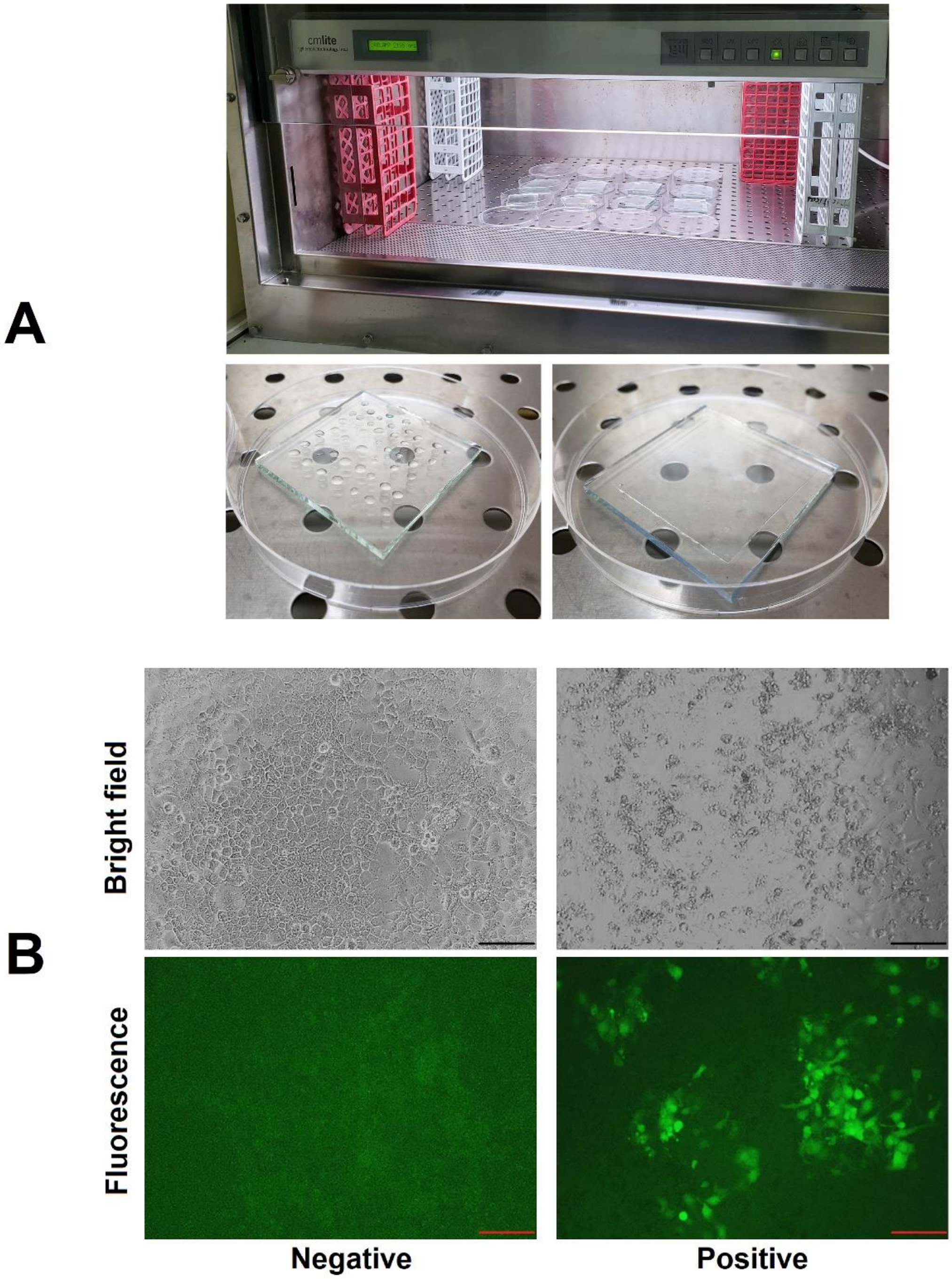

FIGURE S2: Standard curve generated during RT-qPCR amplification of recombinant Human coronavirus 229E/GFP genomic RNA extracted from virions purified through sucrose-cushion ultracentrifugacion. A QuantStudio 5 Real Time PCR thermocycler (Applied Biosystems) was used, and each RNA dilution was analyzed in triplicate.

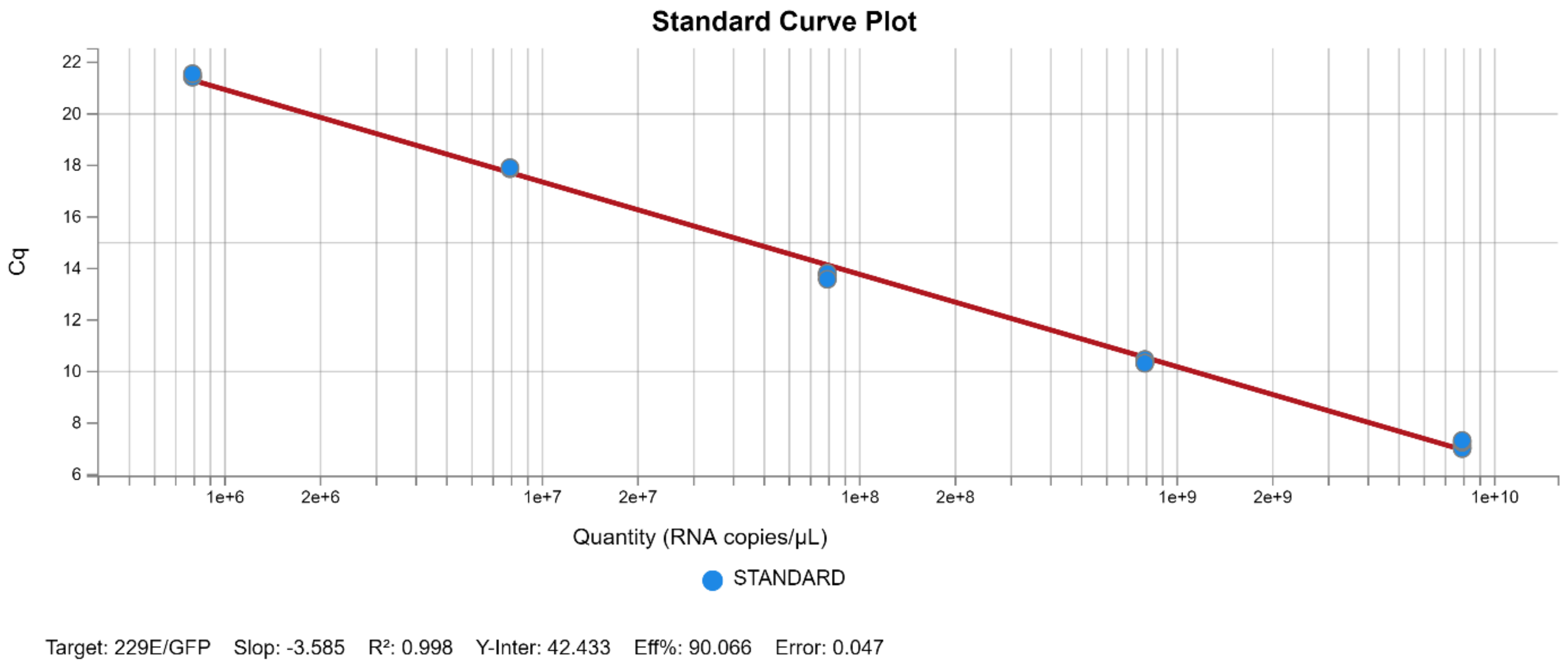

Supplement: SUPPLEMENTAL FILE 1 — Supplemental material. Download spectrum.00269-22-s001.pdf, PDF file, 0.4 MB [file spectrum.00269-22-s001.pdf]
